# Novel genotype-phenotype and MRI correlations in a large cohort of patients with *SPG7* mutations

**DOI:** 10.1212/NXG.0000000000000279

**Published:** 2018-10-24

**Authors:** Channa A. Hewamadduma, Nigel Hoggard, Ronan O'Malley, Megan K. Robinson, Nick J. Beauchamp, Ruta Segamogaite, Jo Martindale, Tobias Rodgers, Ganesh Rao, Ptolemaios Sarrigiannis, Priya Shanmugarajah, Panagiotis Zis, Basil Sharrack, Christopher J. McDermott, Pamela J. Shaw, Marios Hadjivassiliou

**Affiliations:** From the Academic Directorate of Neurosciences (C.A.A.H., R.O'.M., M.K.R., S.P., Z.P., S.B., C.J.M., P.J.S., M.H.), Sheffield Teaching Hospitals NHS Foundation Trust, Royal Hallamshire Hospital; Sheffield Institute for Translational Neuroscience (SITraN) (C.A.A.H., R.S., T.R., C.J.M., P.J.S., M.H.), University of Sheffield; Sheffield Diagnostic Genetics Service (N.J.B., J.M.), Sheffield Children's NHS Foundation Trust; Department of Clinical Neurophysiology (G.R., P.S.), Sheffield Teaching Hospitals NHS Foundation Trust, Royal Hallamshire Hospital; Academic Unit of Radiology (N.H.), University of Sheffield, Royal Hallamshire Hospital; and Sheffield NIHR Biomedical Research Centre for Translational Neuroscience (C.A.A.H., N.H., R.S., P.S., S.B., C.J.M., P.J.S., M.H.), United Kingdom.

## Abstract

**Objective:**

To clinically, genetically, and radiologically characterize a large cohort of *SPG7* patients.

**Methods:**

We used data from next-generation sequencing panels for ataxias and hereditary spastic paraplegia to identify a characteristic phenotype that helped direct genetic testing for variations in *SPG7*. We analyzed MRI. We reviewed all published *SPG7* mutations for correlations.

**Results:**

We identified 42 cases with biallelic *SPG7* mutations, including 7 novel mutations, including a large multi-exon deletion, representing one of the largest cohorts so far described. We identified a characteristic phenotype comprising cerebellar ataxia with prominent cerebellar dysarthria, mild lower limb spasticity, and a waddling gait, predominantly from a cohort of idiopathic ataxia. We report a rare brain MRI finding of dentate nucleus hyperintensity on T2 sequences with *SPG7* mutations. We confirm that the c.1529C>T allele is frequently present in patients with long-standing British ancestry. Based on the findings of the present study and existing literature, we confirm that patients with homozygous mutations involving the M41 peptidase domain of *SPG7* have a younger age at onset compared to individuals with mutations elsewhere in the gene (14 years difference, *p* < 0.034), whereas c.1529C>T compound heterozygous mutations are associated with a younger age at onset compared to homozygous cases (5.4 years difference, *p* < 0.022).

**Conclusions:**

Mutant *SPG7* is common in sporadic ataxia. In patients with British ancestry, c.1529C>T allele represents the most frequent mutation. *SPG7* mutations can be clinically predicted by the characteristic hybrid spastic-ataxic phenotype described above, along with T2 hyperintensity of the dentate nucleus on MRI.

Hereditary spastic paraplegia (HSP) and hereditary cerebellar ataxias (HCA) are heterogeneous groups of progressive neurodegenerative conditions with considerable overlap.^1,2^ HSP is complicated when features such as ataxia, neuropathy, optic atrophy, and weakness are present.^3^ HCA can also be associated with spastic paraplegia. There are over 80 different genetic loci associated with HSP and similar number associated with cerebellar ataxias.^4–6^ This extensive genetic heterogeneity together with the overlapping features of HCA and complicated HSP often causes difficulties in disease classification and clinical approach to genetic diagnosis.

Next-generation sequencing (NGS) gene panels are available for both HSP and HCA patients. However, such panel tests are expensive and not always readily available. Our objective was to describe clinical, genetic, and radiologic features of a British cohort of *SPG7* cases where the phenotype may be helpful in providing guidance to targeted genetic testing. We highlight the importance of such clinical characterization through our experience of diagnosing a large cohort of patients with mutations in the *SPG7* gene, implicated in both HSP and HCA.^7^ In addition, by reviewing all published *SPG7* mutation data, we make important new genotype-phenotype correlations.

## Methods

### Patient cohorts

We studied all cases positive for *SPG7* mutation in our HSP and ataxia cohorts, which mainly include patients from the North of England (cohort study) and analyzed all clinical, genetic, and neuroimaging data.

### Standard protocol approvals, registrations

Patient consent was obtained for genetic testing in accordance with the departmental regulations. Healthy control cases for MRI were recruited as per ethics committee approval (REC reference 09/H1310/79, IRAS 26259). STROBE checklist for cohort study adhered in reporting the data.

### Genetic testing

Libraries of sheared genomic DNA corresponding to panels of either HCA or HSP genes captured using a SureSelect XT custom designed probe set (Agilent, Cheadle, UK), and pair-end sequenced using a HiSeq 2500 instrument (Illumina) was used. Raw data were analyzed using the Genome Analysis ToolKit,^8^ (Broad Institute, Cambridge, MA) according to guidelines.^9,10^ After initial identification of 11 patients with *SPG7* mutations using the ataxia and HSP gene panels, we evaluated the phenotype to identify a triad of spastic paraplegia (usually mild), cerebellar ataxia (with prominent cerebellar dysarthria), and waddling gait indicative of proximal muscle weakness. Thereafter, the majority of patients who presented who had the above triad were analyzed by bidirectional Sanger sequencing and dosage analysis (multiplex ligation-dependent probe amplification kit P213-B1 and B2, MRC-Holland) of all 17 exons of the *SPG7* gene. The remainder of the cohort were identified using either HSP or HCA gene panel testing as before.

Chromatographs were analyzed using Mutation surveyor v4.0.8 (softgenetics.com). Annotation of mutations was carried out in accordance with Human Genome Variation society nomenclature (hgvs.org/mutnomen), with nomenclature based on the reference sequence NM_003119.3. Novel variants in the *SPG7* gene were assessed for pathogenicity using Alamut Visual version 2.9.0 (Interactive Biosoftware, Rouen, France) and prediction software (Provean, MutPred, SNPS & GO and PolyPhen2). Allele frequencies for novel variants in normal control populations were obtained from the Genome Aggregation Database (gnomAD).^11^

### Neuroimaging

MRIs, available for all patients who underwent MRI, were analyzed for cerebellar atrophy. Further subanalysis of the dentate nucleus was undertaken for all patients who underwent brain imaging on the same 3-T MR scanner (Ingenia, Philips Medical Systems, Eindhoven, The Netherlands) using the same T2-weighted sequence (avoid machine-related variability) (cases: n = 21 and controls: n = 16). This was compared with age- and sex-matched controls imaged with this sequence. The axial T2-weighted parameters were as follows: repetition time 3,000 ms, time to echo 80, echo train length 15, number of averages was 1 and 4 mm thick, 512 × 512 matrix. Matching criteria for healthy controls were age within 3 years and sex. Relative signal intensity of the dentate nucleus was compared to normal-appearing pontine white matter and red nucleus. A region of interest (area 20 mm^2^) in these structures was placed in the region of the dentate nucleus with the lowest signal. The dentate nucleus signal was then dichotomized by whether the ratio of the signals was less than or more than 1 (i.e., hypointense or hyperintense compared to normal-appearing white matter in the pons).

### Literature review

**Figure 1:**
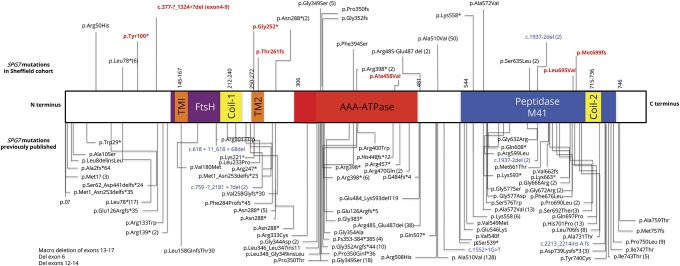
Schematic diagram of the SPG7 protein with important functional domains and positioning of mutations in the Sheffield cohort and all the published pathogenic mutations in the SPG7 gene Mutations described in our cohort of patients are annotated above the SPG7 protein structure, while previously published mutations are below. Allelic frequency is noted within parenthesis. New mutations detected in our cohort are highlighted in red font. Variations denoted in blue are matching complementary DNA sequence of the reported mutations. Some large exon deletions reported are indicated in the text box. Parentheses from mutations removed to create space. AAA = ATPases associated with diverse cellular activities; Coil1 and Coil2 = coiled domain; FtsH = filamentation temperature-sensitive mutant in *Escherichia coli* domain; TM1 and TM2 = transmembrane domain 1 and 2. Reference sequence: NM_003119.3.

Two clinicians independently reviewed clinical and genetic details of all *SPG7* cases thus far reported in the literature (until September 30, 2017). We searched the following terms in PubMed, MEDLINE, Web of Science, and Embase: *SPG7*, paraplegin, hereditary spastic paraparesis, HSP (mutations), spastic ataxia, and ataxia, and selected all the articles reporting *SPG7* and/or paraplegin mutations and reviewed the phenotype and genotype data published. We excluded publications that were not in English or where English translation was not available and articles that did not describe clinical features. All mutations described by us and previously reported are depicted in a schematic diagram in relation to functionally important domains (figure 1) (e-table 1, links.lww.com/NXG/A89).

### Statistical analyses

Statistical analysis was performed using Prism GraphPad V7.0b and SPSS (2015) statistical software programs. One-way analysis of variance was used for multiple group comparisons, and independent samples *t* test and χ^2^ test were used to compare 2 groups.

### Data availability

All anonymized data can be shared on a collaborative basis.

## Results

### Characterization of the phenotype

We identified a total of 42 cases positive for pathogenic mutations in both alleles of the *SPG7* gene (table 1). Initially, 11 cases were identified using ataxia or HSP NGS gene panels (4 patients using ataxia panel and 7 patients using the HSP panel). On reviewing the phenotype of these 11 cases, we noted that 9 individuals had cerebellar ataxia with prominent slurring of speech, mild spasticity, and proximal muscle weakness resulting in a waddling gait.

**Table 1:**
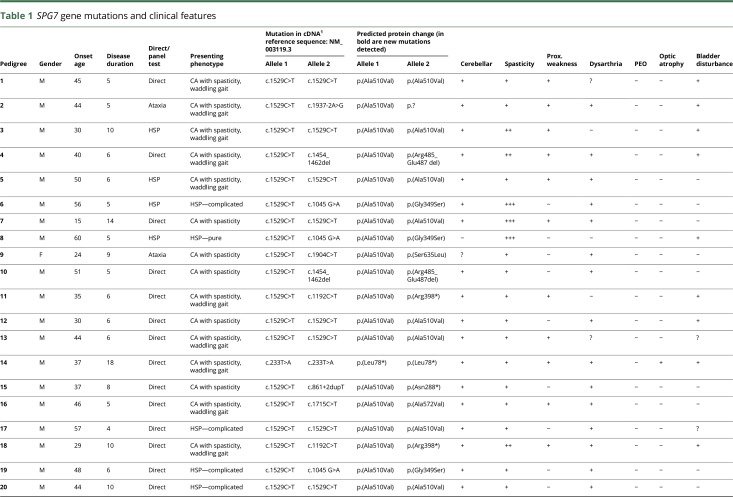

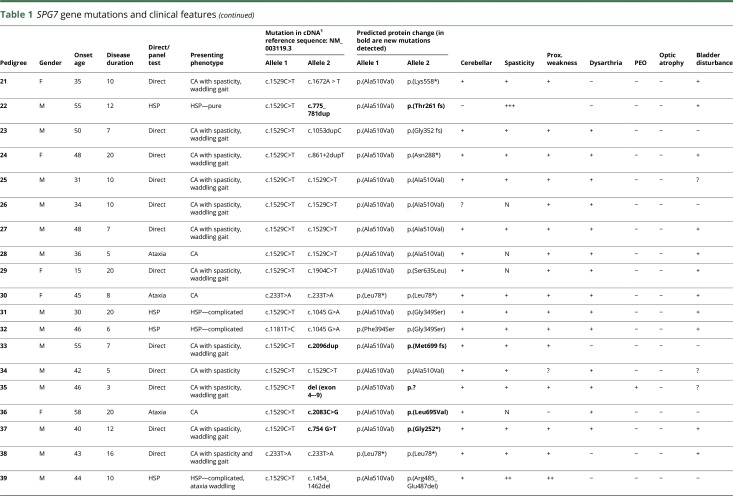

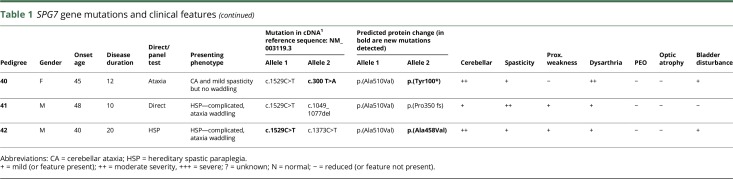
*SPG7* gene mutations and clinical features

### Direct genetic screening based on the phenotype

Following the clinical characterization of the initial 11 patients, we undertook direct testing for mutations in the *SPG*7 gene in patients who demonstrated the above phenotype in a cohort of patients attending the Sheffield Ataxia Centre and HSP clinics. We identified a further 27 cases with pathogenic mutations (table 1). Four other cases were already diagnosed when referred to us (panel tests).

The clinical characteristics of the 42 probands are summarized in table 2. There was no history of consanguinity. Eighty-three percent were male. The average age of symptom onset was 41.7 years (SD: 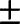
11, median age 44 years). Female patients developed symptoms on average 4 years earlier than male patients (38.5 vs 42.5 years) (table 2). The mean duration of disease at the time of diagnosis was 9.6 years (SD: ±5.2, mode 5). Thirty-eight of 42 patients (90%) were of long-standing British ancestry. Four were UK citizens of Indian, Iranian, German, and Bulgarian descent.

**Table 2:**
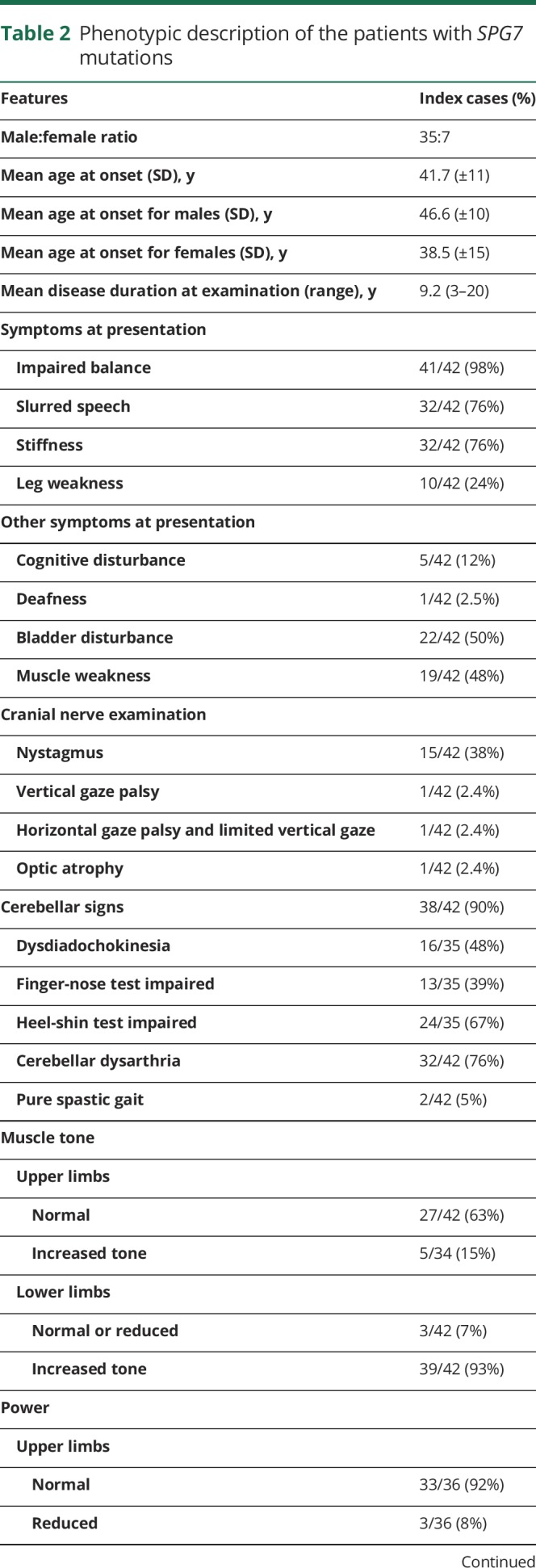

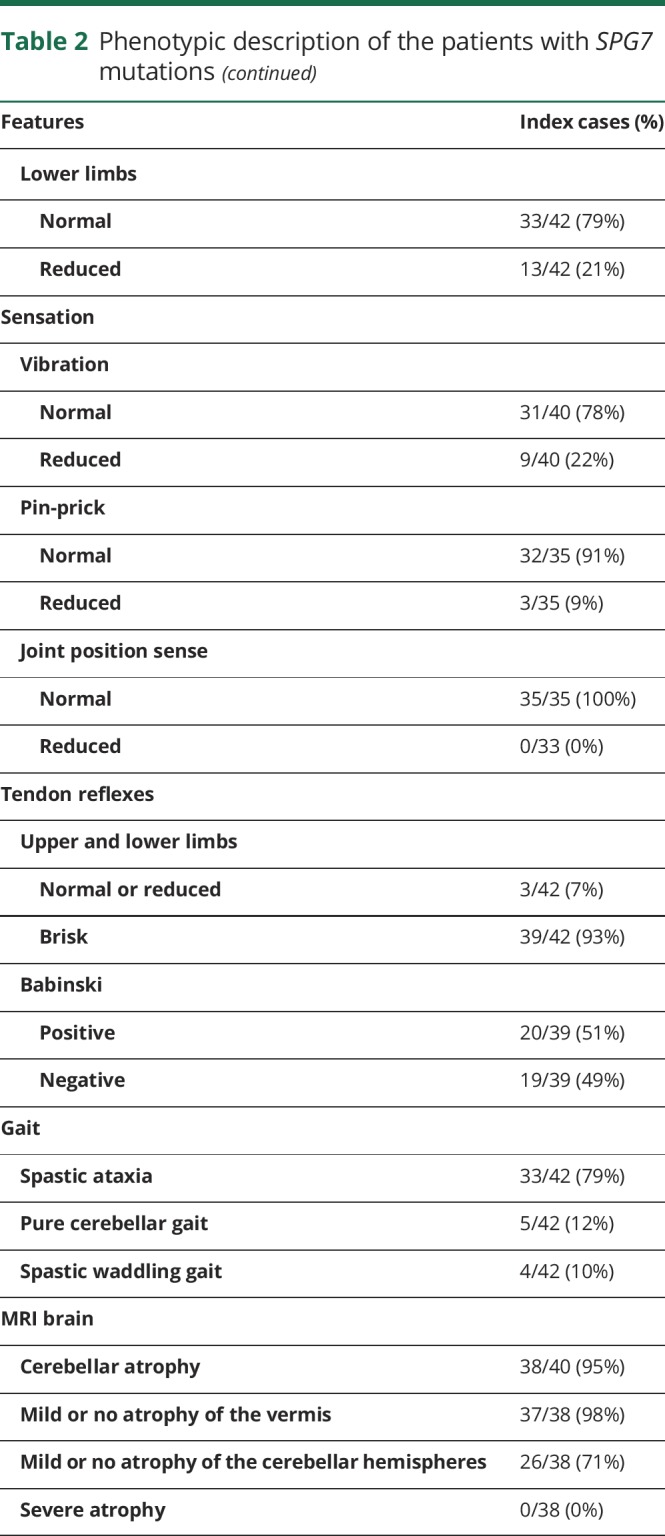
Phenotypic description of the patients with *SPG7* mutations

Ninety-eight percent of cases presented with gait unsteadiness followed by dysarthric speech (76%). Thirty-two patients (76%) complained of mild spasticity. Two patients presented with the typical spastic gait characteristic of HSP (5%) and 7 patients (16%) presented with moderate-to-severe spasticity. Seventy-six percent (32 of 42) had mildly increased lower limb tone and 93% had brisk reflexes, while the Babinski sign was positive in 51%.

At baseline, 38 patients (90%) were found to have at least some evidence of cerebellar ataxia and 33 (79%) were found to have both mild spasticity and cerebellar ataxia. Despite the cerebellar features, only 2 cases were nonambulant, with a total symptomatic disease duration of 399 patient-years. None of the patients could run, and 78% of the cases were using walking aid. The severity of the ataxia was less (median scale for the assessment and rating of ataxia [SARA] score 8, range: 3.5–13.5) when compared to spino cerebellar ataxia 6 (median score 15.0) for the same duration of symptoms.

Sixty-four percent of cases demonstrated the triad of cerebellar ataxia with dysarthria, spasticity, and waddling gait at presentation, and 9 others developed the full clinical picture during follow-up (totaling 87%). Progressive external ophthalmoplegia (PEO) was observed only in 1 patient. Another patient had vertical gaze palsy. Nystagmus was present in 38% of patients. Optic atrophy was seen in 1 patient. Waddling gait was seen in 87% of our cases.

Although 4 patients were found to have reduced vibration sense and 3 had reduced pinprick sensation on clinical examination, none of the 19 patients who underwent neurophysiologic assessment had evidence of large fiber peripheral neuropathy or myopathy.

### Mutation analysis

Fifteen cases (36%) were homozygous for mutations in the *SPG7* gene, while 27 cases (64%) were compound heterozygous. Twelve of the 15 homozygous cases had the common missense mutation in exon 11, c.1529C>T, p.(Ala510Val), while the other 3 cases were homozygous for the c.233T>A, p.(Leu78*) nonsense mutation. Ninety percent of our cases that carried the common mutation p.(Ala510Val) in at least one allele were of British ancestry. The 3 patients homozygous for the p. (Leu78*) nonsense mutation were second-generation British citizens of Indian, Iranian, or Bulgarian descent. The fourth case, of German descent, was compound heterozygous for the c.1181T>C, p.(Phe394Ser) and c.1045 G>A, p.(Gly349Ser) mutations.

The frequency of the c.1529C>T, p.(Ala510Val) mutation in our cohort was 60% (50 of 84 alleles assessed). The second most common mutant allele, c.233T>A, p.(Leu78*), was seen in 3 patients in the homozygous state, while c.1045 G>A, p.(Gly349Ser) was seen in 5 cases in a compound heterozygous state. p.(Ala510Val) and p.Arg485_Glu487del mutations were observed in two-thirds of the disease alleles (50 of 84). In addition, to the single case with a large deletion, several small insertions, duplications, deletions, and splice site mutations were detected on 7 alleles, 5 of which have been previously described. Most of the pathogenic alleles were missense mutations (63 of 84) while 21 were nonsense mutations (table 1).

### Novel mutations in *SPG7*

We discovered 7 novel likely pathogenic mutations in the *SPG7* gene (table 1), of which 5 were null mutations, with 2 frame-shift mutations c.775_781dup p.(Thr261 fs) and c.2096dup p.(Met699 fs), 2 nonsense mutations c.754G>T, p.(Gly252*) and c.300T>A, p.(Tyr100*), and a large deletion encompassing at least exons 4 to 9 (c.377−?_1324+?del) was identified using multiplex ligation-dependent probe amplification. One of the novel missense mutation, c.1373C>T, p.(Ala458Val), results in substitution of a conserved amino acid, which is proven to be deleterious using in silico analysis while found in one allele in gnomAD database supporting pathogenicity. The second missense mutation c.2083C>G, p.(Leu695Val) resulted in substitution of the same amino acid as a previously reported pathogenic mutation c.2084T>C, p.(Leu695Pro).^12^ Predictions by PROVEAN (deleterious), PolyPhen2 (probably damaging), and MutPred (actionable hypothesis) suggested likely pathogenicity, but this was not supported by SNPS & GO (neutral). This allele is present in the East Asian gnomAD normal control population at a frequency of 0.4626%.

### MRI brain

**Figure 2:**
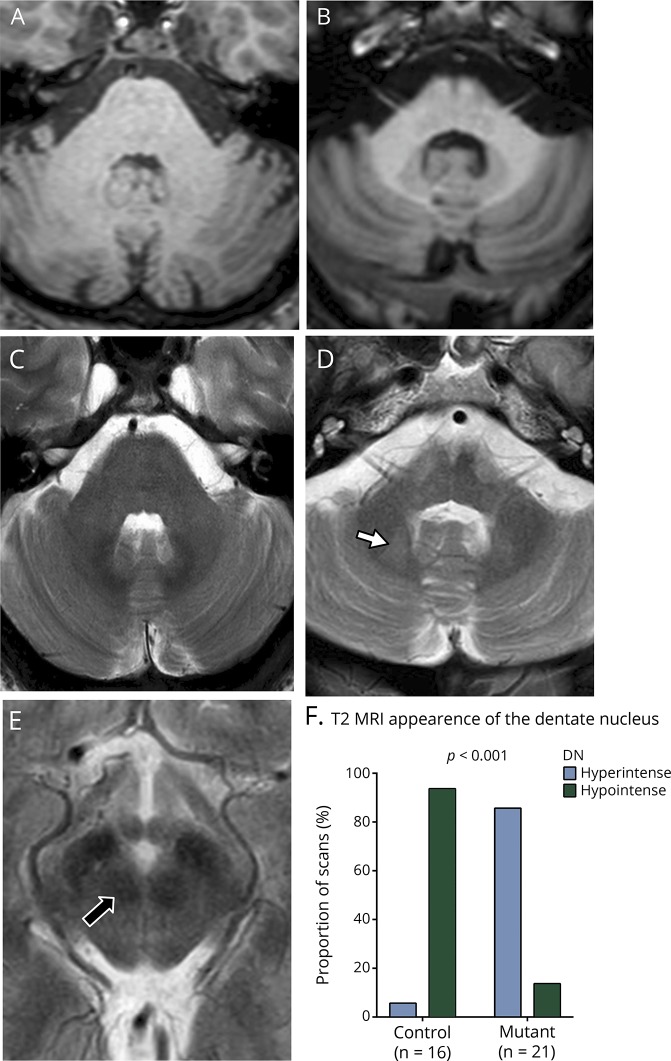
MRI of the brain in SPG7 cases shows T2 hyperintensity of the dentate nucleus (A) T1 axial image across the dentate nucleus (DN) of a control case. (B) T1 axial section through the DN in a patient with c.1529C>T homozygous mutation. (C) T2-weighted axial image of the same control and (D) T2 axial section through DN in the same patient with c.1529C>T homozygous mutation, which demonstrates hyperintense DN (solid white arrow) compared to the normal-appearing white matter. (E) T2-weighted axial image of the same patient, which demonstrates the red nucleus (RN). The RN appears hypointense compared to normal-appearing white matter in all *SPG7* and control cases (solid black arrow with white border). (F) The observation of hyperintense T2 signal of the DN was significantly more frequent in the SPG7 patients compared to the control cases (*p* < 0.001, χ^2^ test value 25.7649).

MRI brain imaging was available in 40 cases. Cerebellar atrophy was noted in 95%, mostly mild atrophy of the vermis (table 2; figure 2, A and B). T1 sequences of both dentate nuclei (DN) and the red nuclei (RN) were not distinguishable between controls and *SPG7* cases (figure 2, A and C). The same T2 sequence on 3-T imaging was available in 21 patients and these were matched with 17 healthy controls. In the 16 healthy controls, the DN were hypointense compared to normal-appearing white matter (figure 2C), and 1 healthy control had DN isointense relative to normal-appearing white matter. The DN were isointense or hyperintense compared with normal-appearing white matter (T2 imaging) in 18 of the 21 *SPG7*-positive cases (figure 2D). Both controls and patients showed no difference in the appearance of the RN, which were hypointense compared to normal-appearing white matter in the pons (figure 2E). The increase in DN T2 hyperintensity on MRI in *SPG7* cases was significant compared to the controls (χ^2^ test value 25.76, at *p* < 0.001) (figure 2F).

### Genotype-phenotype correlation from current and other studies

**Figure 3:**
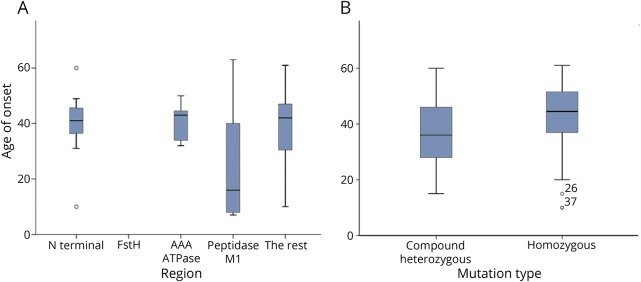
Genotype-phenotype correlation in SPG7 mutations and age at onset of symptoms (A) Association of the position (by the functionally important regions) of the mutation and the age at onset in homozygous *SPG7* cases. N terminal = up to first 140aa; FstH = 141-250aa; AAA ATpase = 306-481aa; M41 peptidase = 544-746aa; the rest = mutations in any other area(s), which is/are not described as above. We selected homozygous cases because of the uniformity they create by harboring 2 similarly, mutated alleles, to compare the effect of the mutation within functionally important domains of the SPG7 protein on the age at onset. One-way analysis of variance with multiple comparisons and post hoc Tukey test showed a significantly (*p* = 0.034) younger age at onset (14.63 years, SE 5.25, 95% confidence interval: 0.82–28.4) for those with homozygous mutations in the M41 peptidase domain compared to a mutational position in a functionally undefined domain (“the rest”). (B) The c.1529C>T common mutation when in the homozygous state is associated with a significantly later age at onset than when in the compound heterozygous state. (C)1529C>T patients provide a degree of mutational homogeneity, in that at least 1 allele is constant allowing comparison between homozygous and compound heterozygous states. Compound heterozygotes developed symptoms on average 5.4 years earlier than the c.1529C>T homozygotes (*p* = 0.022, independent samples *t* test for equality of mean values with equal variances assumed).

We analyzed mutations in the different functionally important domains of *SPG7* shown in figure 1 for any impact on age at onset of symptoms. Patients who had homozygous mutation in the M41 peptidase domain had an earlier onset of disease symptoms (by 12 years) compared to patients with mutations in a nonfunctionally assigned domain (*p* < 0.022) (figure 3A). Having homozygous, compound heterozygous mutations or the presence of null alleles did not have an impact on age at onset. However, we also noted that patients with the c.1529C>T mutation when in a compound-heterozygote state developed symptoms 8 years earlier compared to c.1529C>T homozygous cases (*p* < 0.019, unpaired *t* test) (figure 3B).

## Discussion

We describe a large British cohort of 42 unrelated and previously unreported cases with mutations in the *SPG7* gene. The largest other single-center cohort so far reported is a Dutch cohort of 46 unrelated families.^13^ We propose that the phenotype of cerebellar ataxia (with marked dysarthria), mild lower limb spasticity, and waddling gait is clinically distinct and should alert clinicians to direct genetic testing for *SPG7*. Such an approach identified 64% of our cohort. While *SPG7* biallelic mutations have historically been associated with HSP, it is now clear that ataxia is the major clinical presentation, as only 26% of our cohort presented with an HSP-like phenotype.^7,14,15^ In another UK-based study, *SPG7* accounted for 18.6% of 70 patients with unexplained ataxia and pyramidal signs.^7^ *SPG7* is the fourth commonest cause of any genetic ataxia in the United Kingdom and the second commonest recessive ataxia.^16^ In support of this finding, 90% of our *SPG7* cohort demonstrated gait ataxia with cerebellar dysarthria (table 2).

Only 2 patients were wheelchair-dependent, indicating that ambulatory loss appears to be rare in *SPG7* cases,^15^ with an average SARA score of 8 (range: 3.5–14). This favorable prognostic factor will be useful when counseling *SPG7* patients and their families.

A considerable proportion of our cohort was male (83%). Female patients tended to develop symptoms about 4 years earlier. The median age at onset of symptoms was 44 years, indicating that *SPG7*-related disease is a late-onset disease in keeping with previous reports.^13,17^ The age at onset, however, did range between 15 and 60 years; therefore, *SPG7* can rarely present with an early-onset ataxia. The recessive inheritance accounts for the lack of a positive family history. Absence of a family history should therefore not deter clinicians from *SPG7* testing.

PEO was only seen in one of our patients but has been reported in 11% of *SPG7* cases worldwide. A previous report found that 13% of the patients with PEO have *SPG7*. PEO was also reported in 1 of 5 cases in a UK cohort of complex HSP.^18^ Overall, PEO in *SPG7* was also rare in other cohorts, including a French (2/23),^19^ a Dutch (2/46),^13^ and a French Canadian cohort (none of the 22 individuals).^15^ A longitudinal study of *SPG7* patients from the United Kingdom reported a median follow-up duration of 23 years from presentation to detecting PEO,^7^ as did a Norwegian group (median follow-up of 24 years).^20^ Clinicians should be aware that PEO-like features in SPG7 are rare but can develop late in the disease process.

Optic neuropathy was reported in 9.5% of the worldwide *SPG7* cases, compared to one patient in our cohort. In a French cohort of *SPG7* patients, 44% had evidence of optic neuropathy based on optical coherence tomography, yet 40% of the patients with optic neuropathy had normal-appearing optic discs on funduscopy. It is therefore likely that optic atrophy is common in *SPG7*-mutated patients but the clinical significance of this remains unclear.

*SPG7* cases have mild cerebellar atrophy and none had severe atrophy (table 2). The increased T2 signal from the dentate nucleus in *SPG7* cases compared to controls has not been previously reported. The dentate nucleus is a site of iron accumulation in normal aging, and this is usually associated with reduced T2 signal. The high signal noted in *SPG7* cases does not appear to be due to a globally reduced brain iron accumulation. In support of the MRI findings are postmortem data from an *SPG7* case, which showed neuronal loss in the dentate nucleus.^21^ While the above imaging finding is not specific for *SPG7* mutations, yet it is an important characteristic and merits further consideration.^22^ We propose that dentate nucleus hyperintensity on MRI T2 sequences, without severe overlying cerebellar atrophy and in the context of a typical phenotype, aid the diagnosis of mutant *SPG7.*

We also discovered 8 cases heterozygous for *SPG7* (*SPG7*-Het) (50% were c.1529C>T). As the pathogenicity of the *SPG7*-Het is not well established, we have not included further analysis. However, analysis of the MRI appearances showed 2 of 8 *SPG7*-Hets had T2 hyperintensity of the DN (e-table 2, links.lww.com/NXG/A90).

The association of waddling gait has not been previously highlighted in relation to *SPG7*. There are a number of reports describing muscle weakness over and above the mild pyramidal weakness seen in patients with HSP.^19^ In keeping with our observations, myopathic features were noted in a PEO cohort.^17^ Furthermore, two-thirds of the *SPG7* cases from the Dutch cohort were noted to have lower limb muscle weakness.^13^ This weakness may account for the rationale of performing muscle biopsies in some cases that have, on occasions, shown evidence of mitochondrial dysfunction.^14,19,23,24^

More than 242 cases of *SPG7* have been described worldwide. Analysis of the mutations demonstrates unique genotype-phenotype correlations in *SPG7*, wherein mutations in the M41 peptidase domain are associated with younger age at onset and c.1529C>T homozygous mutations tend to associate with later onset of disease compared to compound heterozygotes. Further studies are needed to confirm above findings.

We identified 7 novel mutations, 70% resulting in premature truncation of the paraplegin protein. The c.1529C>T mutation was present in at least one allele in all patients with British ancestry, strongly supporting a previous report of its association with patients with British heritage.^14^ We observed that c.1529C>T is the commonest mutant *SPG7* allele worldwide, and this allele frequency in our cohort was 60%. The c.1454_1462del mutation is the second most common mutation (9%).

We have highlighted that *SPG7* is a common cause of sporadic ataxia. We recommend direct genetic testing for *SPG7* mutations when cerebellar ataxia with dysarthria is associated with mild lower limb spasticity and a waddling gait. If the patient is of long-standing British ancestry, directly testing for the c.1529C>T mutation is highly likely to be diagnostic. The MRI feature of relative T2 hyperintensity of the DN is also strongly supportive of mutant *SPG7*.

## Glossary

DN: dentate nuclei
HCA: hereditary cerebellar ataxias
HSP: hereditary spastic paraplegia
NGS: Next-generation sequencing
PEO: Progressive external ophthalmoplegia
RN: red nuclei
SARA: Scale for the assessment and rating of ataxia
